# The Impact of Postmastectomy Radiation Therapy on Immediate Prepectoral Reconstruction with Polyurethane-Coated Implants

**DOI:** 10.1007/s00266-025-05119-w

**Published:** 2025-08-21

**Authors:** Marzia Salgarello, Valentina Sara Pino, Federico Taraschi, Giuseppe Visconti, Francesco Cellini, Fabio Marazzi, Valeria Masiello, Liliana Barone Adesi

**Affiliations:** 1 UO Chirurgia Plastica, Dipartimento per la Salute della Donna, del Bambino e di Sanità Pubblica - Fondazione Policlinico Universitario “Agostino Gemelli” IRCCS - Università Cattolica del “Sacro Cuore” - Largo A. Gemelli 8, 00168 Rome, Italy; 2 Università Cattolica del “Sacro Cuore” - Largo A. Gemelli 8, 00168 Rome, Italy; 3 Residency Program in Plastic Surgery, Università Cattolica del “Sacro Cuore” - Largo A. Gemelli 8, 00168 Rome, Italy; 4 UOC Radioterapia Oncologica, Dipartimento di Diagnostica per Immagini, Radioterapia Oncologica ed Ematologia… - Fondazione Policlinico Universitario “Agostino Gemelli” IRCCS - Università Cattolica del “Sacro Cuore” - Largo A. Gemelli 8, 00168 Rome, Italy

**Keywords:** Prepectoral breast reconstruction, Adjuvant radiotherapy, Breast reconstruction, Polyurethane implants

## Abstract

**Background:**

Increasingly popular, prepectoral breast reconstruction preserves the pectoralis major muscle’s anatomy and function. Although polyurethane-coated implants in this context have yielded encouraging results, their interplay with postmastectomy radiation therapy (PMRT) is not well-documented, particularly considering PMRT's known adverse effects on implant-based reconstructions. The study aimed to evaluate the aesthetic outcomes and radiation therapy (RT) damage in patients undergoing prepectoral reconstruction with polyurethane-coated implants receiving PMRT, as well as the influence of mastectomy flap thickness on RT side effects.

**Methods:**

In 47 patients receiving immediate breast reconstruction with prepectoral polyurethane-coated implants followed by PMRT, aesthetic results were assessed using the Likert scale, and RT damage was scored with the LENT–SOMA scale. The study retrospectively analyzed the impact of different RT techniques (3D vs. IMRT) and examined the correlation between mastectomy flap thickness and RT adverse effects.

**Results:**

At 12-month follow-up, the mean Likert score for patients treated with IMRT/VMAT was 13.06 (SD: 2.55) compared to 11.79 (SD: 2.37) for those treated with the 3D technique. LENT–SOMA scores were 1.46 (SD: 1.13) for IMRT/VMAT and 3.11 (SD: 1.41) for 3D. A negative linear correlation was found between mastectomy flap thickness and RT damage.

**Conclusions:**

Preliminary findings are favorable for the use of prepectoral polyurethane-coated implants in immediate breast reconstruction with PMRT, particularly when using IMRT and in patients with thicker mastectomy flaps.

Level III, therapeutic study.

**Level of Evidence III:**

This journal requires that authors assign a level of evidence to each article. For a full description of these Evidence-Based Medicine ratings, please refer to the Table of Contents or the online Instructions to Authors www.springer.com/00266.

## Introduction

Currently, prepectoral breast reconstruction (PPBR) has become more and more popular due to its satisfying aesthetic outcomes, low functional detriment, and low complications rate [1, 2]. PPBR has been able to overcome most of the drawbacks of the traditional submuscular reconstructive technique such as unpleasant aesthetic results and postoperative discomfort due to implant displacement, breast animation deformity (BAD), pain associated with muscle spasm, and capsular contracture [3, 4].

Therefore, the interest in prepectoral reconstruction has led, in the last years, to new anatomical researches [5]. Rehnke et al. described the superficial fascia system as a tridimensional closed fascia and fat system that surrounds the corpus mammae, a crucial structure to preserve during a conservative mastectomy [6]. Following this new anatomical knowledge of the superficial fascial system, the concept of “anatomical mastectomy”, which consists in the removal of the entire breast parenchyma saving the overlying skin and subcutaneous tissue, is becoming increasingly popular.

In addition, the thickness of subcutaneous tissue between the dermis and the gland is predictive of skin perfusion of mastectomy’s flaps. The focus is to maintain an adequate thickness of the mastectomy flaps, making sure that the whole tumor is removed. The recurrence rate with this technique is 2.6%, and it is considered oncologically safe [7].

The PPBR can be performed using ADM. Berna et al. described the first case of wrapping the breast implant with a large sheet of ADM to prevent the direct contact of the silicone implant with the surrounding subcutaneous tissues [8]. However, the use of ADM has been reported to increase risks of seroma infection, and it is associated with higher medical costs [9]. Alternatively, it is possible to place micropolyurethane-covered implants in the prepectoral pocket which do not require coverage with ADM, thanks to their peculiar surface, capable of creating a velcro effect. In two-stage submuscular breast reconstruction, the advantages of polyurethane implants have already been extensively validated in terms of implant shape stability and reduction of post-radiotherapy contracture [10]. Since 2019, the possibility of using micropolyurethane-coated implants in the prepectoral site has been introduced, with a reduction in the incidence and degree of capsular contracture compared to DTI reconstruction with textured implant placement [11–15].

Therefore, PPBR with polyurethane-coated implants is becoming increasingly popular with encouraging results, but its interaction with post mastectomy radiotherapy (PMRT) is still unknown.

Radiotherapy is widely used for patients with breast cancer. Radiation acts primarily through creating double-strand DNA breaks and also causes a number of other molecular and epigenetic level effects in both cancerous cells and nearby healthy tissues [16]. There is available evidence that shows how PMRT in high-risk breast cancer patients decreases locoregional recurrence and improves overall survival. Indications include: in pts with ≥ 4 or more positive axillary nodes strongly considered in pts with pN1 ≥T3 stage or invasive disease's margins <1 mm. In addition, also patients with axillary nodal involvement after neoadjuvant systemic therapy should receive PMRT. The breast volume can be irradiated with different modalities: 3D conformal irradiation, i.e., with two fields tangent to the chest wall generated by linear accelerators, or intensity-modulated technique (IMRT) or volumetric arc technique (VMAT) in cases where there may be a benefit from a greater sparing of organs at risk. For about 15 years, IMRT techniques have been introduced in the treatment of breast cancer with the aim of improving target coverage and dose homogeneity that, in the long term, translate into better aesthetic results [17, 18]. Anyhow, the decision to recommend PMRT requires a great deal of clinical judgment [19, 20]. Nevertheless, PMRT is known to increase the risk of multiple adverse outcomes after breast reconstruction. According to the iBAG study, the largest multicenter data collection on prepectoral breast reconstruction carried out only with full-coverage porcine ADM (Braxon®), collecting the experience of 30 hospital (including Policlinico A. Gemelli IRCCS) centers in a 6‐year period, RT appeared to be a statistically significant risk factor for the development of postoperative seroma, capsular contracture, rippling, and implant loss [21]. Many other studies have been conducted on the interaction of RT with ADM, but there are no data regarding the interaction with polyurethane-coated implants [22–25].

Therefore, the aim of this study was to evaluate the aesthetic outcomes and radiotherapy (RT) damage in patients undergoing prepectoral reconstruction with polyurethane-coated implants and PMRT, particularly the effect of RT and the possible correlation between radiation technique applied and outcomes obtained. Additionally, the impact of mastectomy flap thickness on the adverse effects of RT was investigated.

## Materials and Methods

We performed a retrospective clinical analysis of 47 patients who underwent adjuvant radiation treatment after mastectomy and direct-to-implant (DTI) reconstruction with anatomic micropolyurethane-coated implants (Polytech Microthane Sublime Line®, Dieburg, Germany) between September 2016 and October 2021 at our institution. According to preoperative conditions, each patient was informed about the reconstructive options and signed the informed consent.

Baseline measures of demographic–anthropometric variables (age, weight, height, BMI), clinical (smoke, comorbidities, preoperative assessment of the thickness of the gland envelope according to Rancati [26]), surgical (intraoperative measurement of the thickness and perfusion of the mastectomy flap, weight of mastectomy, implant size) and RT-correlated factors (total dose, fractional dose, Clinical Target Volume CTV, technique and method of administration) were collected in the medical records (Table 1).

**Table 1:** Data summary table

No. of patients	47
Mean age, year	44.37 ± 9.58
Mean follow-up, months	12
Mean BMI	25 ± 5.4
Comorbidities	
0	20
1	15
>1	12
Indications	
SSM	3
NSM (Right)	22
NSM (Left)	25
Procedures	
Monolateral	50
Bilateral	3
Mean mastectomy weight	277.30 ± 141.71
Post-mastectomy radiotherapy	47

A BMI greater than 35, active cigarette smoking habit, previous radiation therapy, uncontrolled diabetes mellitus, and chronic immunosuppressive conditions were considered exclusion criteria for increased risk of perioperative complications. A preoperative assessment of the thickness of the gland envelope according to Rancati was performed; through preoperative digital mammography, the thickness of the mastectomy flap could be predicted, and the feasibility of the prepectoral reconstructive technique was confirmed in the context of the multidisciplinary evaluation of breast surgeon, plastic surgeons, and radiologists [26]. Intraoperatively after mastectomy, the near-infrared camera Fluobeam Clinical System^®^ (Fluoptics, Grenoble, France) was used to obtain fluorescence images of mastectomy flap perfusion by indocyanine green (0.25 mg/kg) angiography in 5 ml of double-distilled water [27, 28]. The prepectoral reconstructive option was confirmed when area enhancement was uniform in each area of the mastectomy flaps. Considering the weight of the mastectomy, we used sizers to choose the definitive breast implant that best filled the prepectoral pocket after mastectomy. Anatomical micropolyurethane-coated implants (Polytech Microthane Sublime Line®, Dieburg, Germany) were placed in all patients. Two Blake drains were placed in the upper and lower prepectoral pocket. We applied a compression bra to all patients. After discharge, patients were evaluated weekly during the first month and then every 6 months.

Patients underwent, according to the guidelines and to the multidisciplinary team discussion, to adjuvant RT, either:

- radiotherapy to the chest wall for a total dose of 5040/180 cGy or 5000/200 cGy
- drainage lymph nodes (I–IV axillary level ± I–V intercostal space for the internal mammary lymph nodes) for a total dose of 5040/180 or 5000/200 cGy.

The choice of treatment was based on the clinical and pathological stage as well as the type of surgery performed. Specifically, according to NCCN Breast, AIRO Breast, and ASTRO 2018 guidelines, the purpose of radiotherapy was to reduce the locoregional risk of disease. Radiation treatments were all performed with external beam radiotherapy by 3D conformal or IMRT/VMAT, using photons with an energy range of 6–10–15 MV. Follow-up data collected in the postoperative period were regarding complications in the two groups (patients undergone 3D RT vs. IMRT/VMAT): infection, seroma, hematoma, skin dehiscence and necrosis, capsular contracture, implant exposure, and rippling. Regarding adjuvant radiation treatment, acute RT injury was recorded, and during follow-up (starting 6 months after the end of RT), long-term effects were assessed by the international grading system of late outcomes of radiation therapy: Late Effects in Normal Tissues Subjective, Objective, Management and Analytic scales, LENT–SOMA. The LENT–SOMA system is now universally recognized as the best system for recording the toxic effects of radiotherapy in clinical trials [29–31]. The grading related to the LENT–SOMA scale is assigned on the basis of subjective, objective, and clinical management parameters always by the same operator (Table 2). To simplify the study, only the objective parameters were considered. Finally, an assessment of long-term aesthetic outcomes was made by modified Likert scale. The aesthetic results obtained after PPBR with polyurethane-covered implants and RT were evaluated separately by a group of blinded plastic surgeons, using our standard evaluation method [32]. This evaluation was based on clinical examination and review of clinical breast photographs.

**Table 2:** Data summary table

LENT/SOMA				
Breast	Occasional and minimal hypertension, pruritus	Intermittent and tolerable	Persistent and intense	Refractory and excruciating
Subjective pain				
Objective				
Telangiectasia	< 1 cm^2^	1–4 cm^2^	< 4 cm^2^	
Fibrosis	Barely palpable, increased density	Definite increased intensity and firmness	Very marked density reaction, and fixation	
Edema	Asymptomatic	Symptomatic	Secondary infection	
Retraction, atrophy	10–25%	> 25–40%	> 40–75%	Whole breast
Ulcer	Epidermal only, < 1 cm^2^	Dermal only, > 1 cm^2^	Subcutaneous	Bone erxposed, necrosis,
Lymphedema, arm circumference	2–4 cm increase	> 4–6 cm increase	> 6 cm increase	Useless arm
Skin				
Pigmentation change	Transitory, slight	Permanent marked	–	–

*LENT* Late effect normal tissues task force,* SOMA* Subjective, objective, management and analytic

Statistical analysis was performed using IBM® SPSS® Statistics 20. Considering the absence of distributional normality in the data, as confirmed by the Shapiro–Wilk normality test, we performed the data analysis using nonparametric tests. *P* values less than 0.05 were considered statistically significant. For the analysis of clinical–surgical variables associated with reconstructive outcome, we used Fisher’s exact test and Pearson’s Chi-square to assess the presence of statistically significant differences between the 3D RT group (group A) and IMRT (group B). For the analysis of intraoperative variables (mastectomy weight and flap thickness), and their correlation with the reconstructive outcomes obtained, we used Spearman correlation index R for ranks. Patients were treated according to common clinical practice and in accordance with the current information in the Technical Data Sheet of the devices used and the published scientific literature. This study was conducted in accordance with the principles of Good Clinical Practice and the Declaration of Helsinki.

## Results

**Fig. 1:**
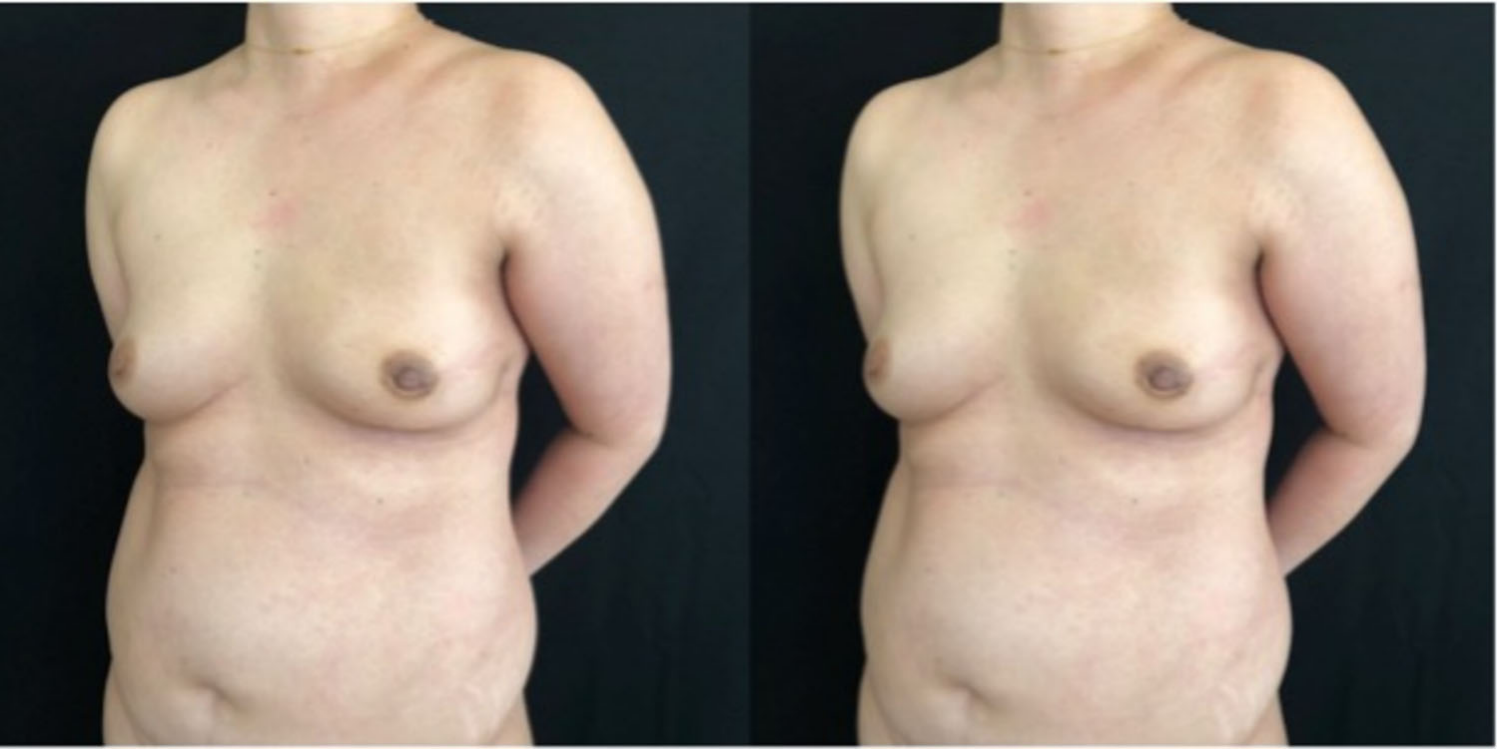
Prepectoral reconstruction with micropolyurethane-coated implant and postoperative radiotherapy. Left: preoperative photograph of a patient with stage 3a ductal carcinoma underwent left NSM and reconstruction with a 175 cc polyurethane-coated implant without contralateral adjustment. Right: follow-up at 12 months. The patient underwent postoperative radiotherapy. LENT–SOMA score = 2. The patient was offered fat grafting surgery to regenerate the tissue and improve the definition of the inframammary fold.

**Fig. 2:**
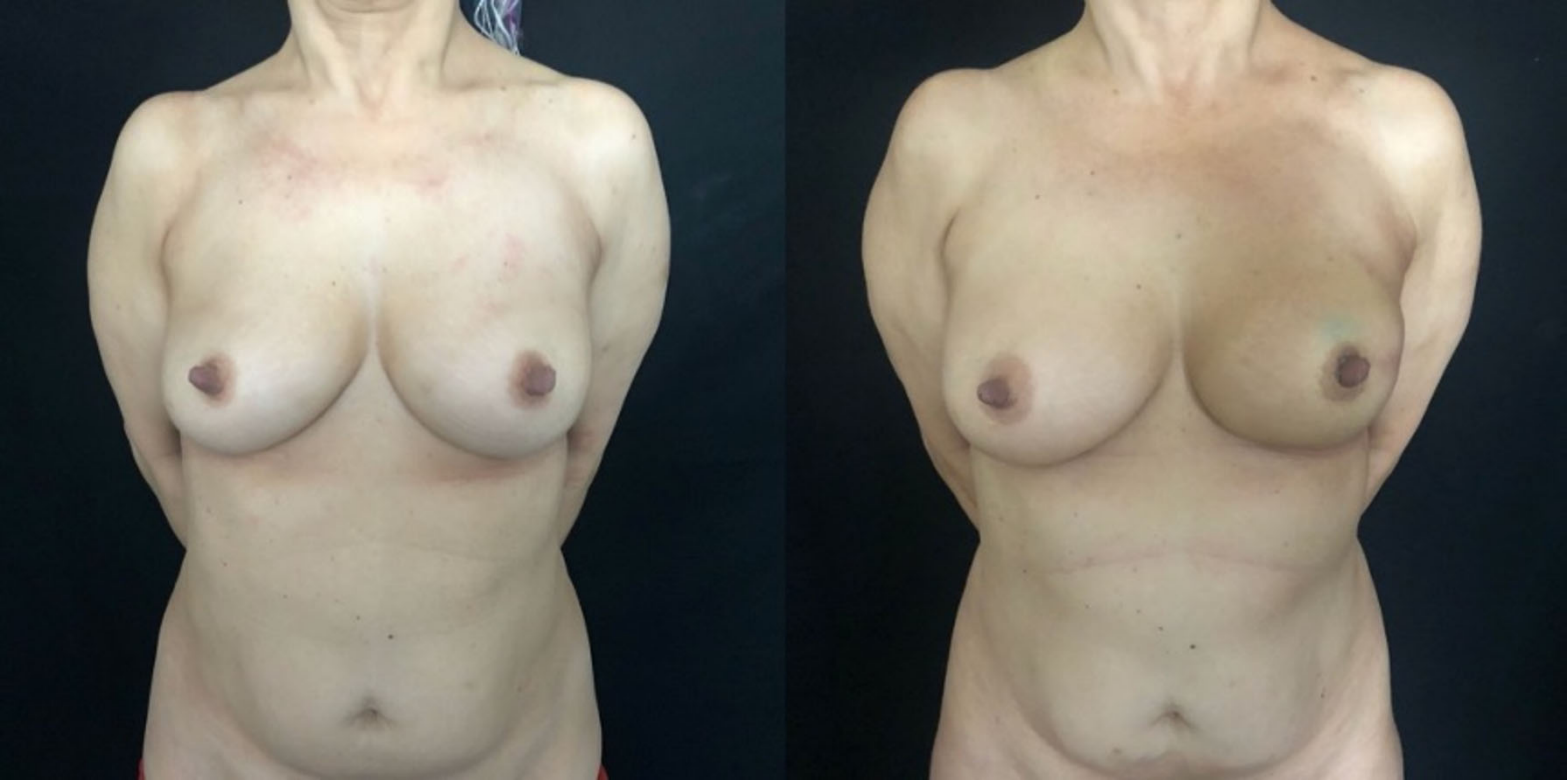
Left: preoperative photograph of a patient with left lobular carcinoma, underwent left NSM (mastectomy weight 190 g and mastectomy flap thickness 1.1 cm) and reconstruction with a 230 cc polyurethane-coated implant without contralateral adjustment. Right: follow-up at 12 months. The patient underwent postoperative radiotherapy with 3D technique. LENT–SOMA score = 3. The patient was offered fat grafting surgery to regenerate the tissue and improve the profile of the inframammary fold.

**Fig. 3:**
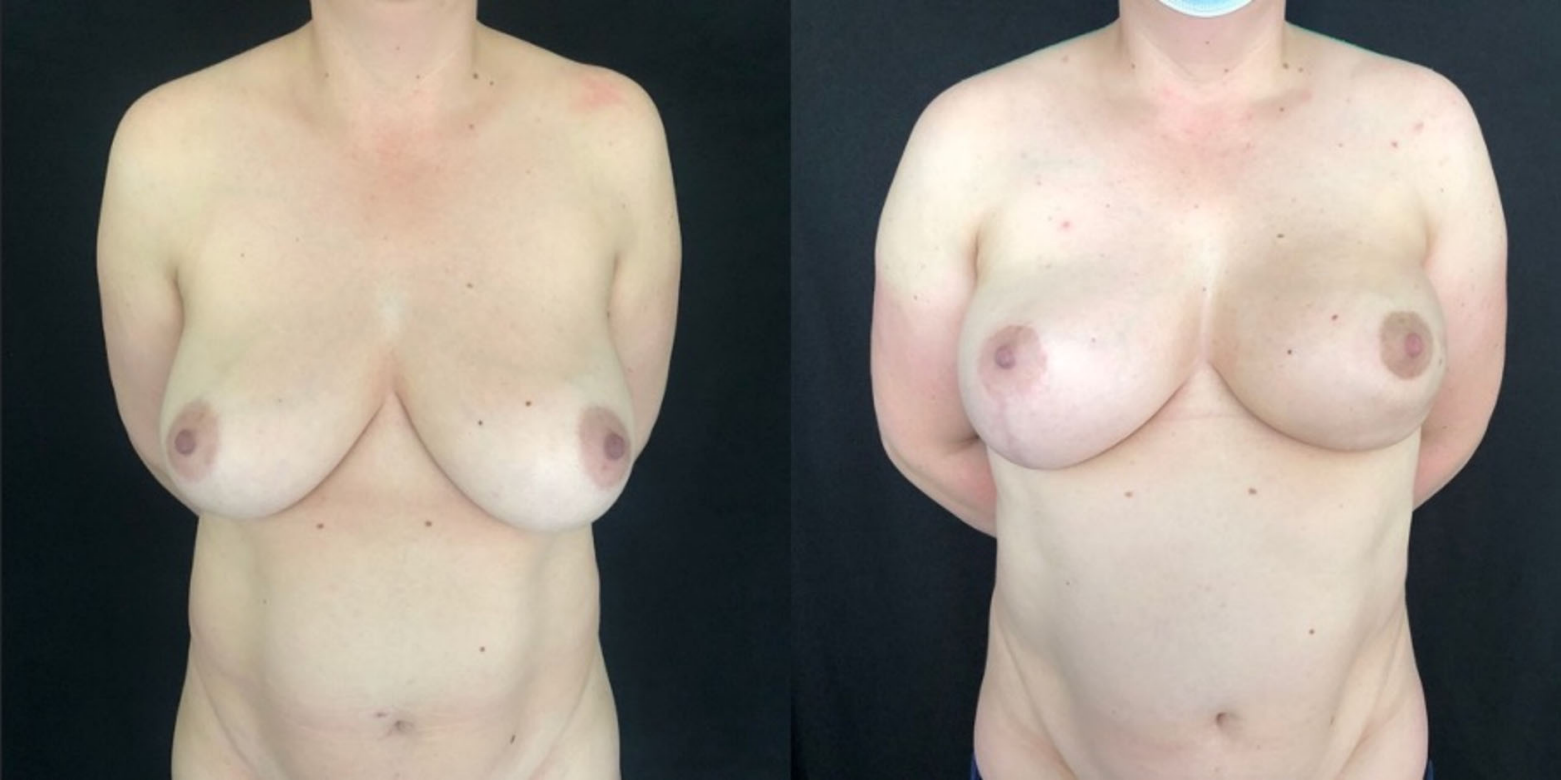
Left: preoperative photograph of a patient with left lobular carcinoma, underwent left NSM (mastectomy weight 420 g and thickness of the mastectomy flap 1.3 cm) and reconstruction with a 420 cc implant covered in polyurethane with mammoplasty J-contralateral scar. Right: follow-up at 12 months. The patient underwent adjuvant radiotherapy with IMRT technique. LENT–SOMA = 1.

**Fig. 4:**
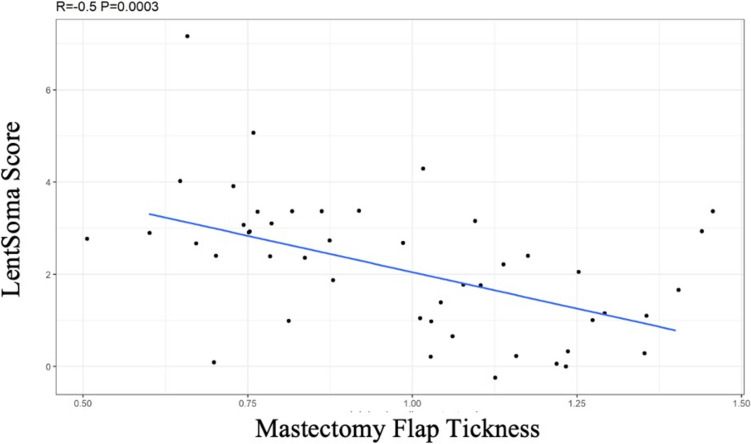
Scatter plot: correlation between LENT–SOMA score and mastectomy flap thickness

Forty-seven patients were included in the study, 3 patients underwent skin sparing mastectomy (SSM), and 44 patients underwent nipple-sparing mastectomy (NSM) and PPBR with micropolyurethane-coated implants. All patients underwent postoperative radiation therapy with a mean follow-up of 12 months (Fig. 1). The mean age of the patients included in the study was 44.37 ± 9.58 with a BMI of 25 ± 5.4. In our sample 2 patients were diabetic, 6 patients reported vascular disease, 7 had hypertension, and 3 had immune disease. A total of 50 mastectomies were performed with an average mastectomy specimen weight of 277.30 g ± 141.71. The average intraoperative mastectomy flap thickness was 0.94 cm ± 0.25. Postoperative complications were divided into early and late (appeared 1 month after surgery); 5 patients experienced early complications such as wound dehiscence (2 patients), seroma (2 patients), and erythema (1 patient); and 6 patients experienced grade III capsular contracture (late complication). Adjuvant radiotherapy with 3D technique was delivered in 38.3% of cases (group A) (Fig. 2), and IMRT was delivered in 61.7% of cases (group B) (Fig. 3). We found a statistically significant difference between the two groups A and B related to acute RT injury (5 cases in group A, *p* value = 0.008). As underlined by Pearson's chi-square test, we found a statistically significant differences between group A and B in the variables related to the Likert scale items, and we found statistical significance for the scar item and for the sum of all items with a *p* value of 0.005. We found no statistically significant differences related to breast volume, breast contour, inframammary sulcus, breast position, projection, breast shape, and overall breast symmetry. We found statistically significant differences (*p* = 0.01) between group A and B related to LENT–SOMA score and single fibrosis parameter. We found no statistically significant differences related to the individual LENT–SOMA score parameters such as atrophy, edema, ulcers, telangiectasia, lymphedema, and pigmentation. At 12-month follow-up, we evaluated and analyzed in the two groups other parameters such as capsular contracture, areas of minus/plus, and rippling, but no statistically significant differences were found. No statistically significant correlation was found between mastectomy flap weight and acute RT injury (*p* = 0.98), capsular contracture (*p *= 0.13), Likert scale (*p* = 0.6), and LENT–SOMA score (*p* = 0.96) with the Spearman's test. No significant differences were found between mastectomy flap thickness, acute RT injury (*p* = 0.98), and postoperative complications (*p *= 0.53). As underlined by Spearman's test, we detected a statistically significant positive correlation between mastectomy flap thickness and LENT–SOMA score shown in the scatter plot (Fig. 4).

## Discussion

The first prepectoral breast reconstruction with preservation of the pectoralis major muscle was described by Snyderman in the 1970s, and it was associated with a high rate of reconstructive failure due to mastectomy flap necrosis (13.5%) and implant exposure (6.7%) [33]. Furthermore, the unsatisfactory aesthetic results, with rippling and visible upper implant profile, represented one of the main problems of this reconstructive approach. The evolution of breast reconstruction techniques led to submuscular reconstruction with implant placement under the pectoralis major muscle in order to obtain an adequate coverage of the implant and to reduce the risk of exposure in case of mastectomy flap necrosis. However, the current scientific literature draws our attention to the consequences of pectoralis major muscle dissection: pain, shoulder joint dysfunction, and breast animation deformity [34–36]. The problems associated with submuscular reconstruction then have led to reconsideration of prepectoral breast reconstruction as a viable reconstructive option [37]. With the progressive improvement in the quality of postmastectomy skin flaps and increasing application of nipple-sparing mastectomy across many centers, there has been a concurrent rise in the rate of muscle-sparing, prepectoral breast reconstruction. As a matter of fact, Franceschini et al. demonstrated that prepectoral immediate prosthetic breast reconstruction (PP-IPBR) can represent a valid alternative to traditional submuscular IPBR, improving outcomes and patient quality of life; it is easier to perform, reduces operative time, and minimizes complications related to manipulation of the pectoralis major muscle, while also contributing to the containment of costs [38]. In 2019, Roy De Vita et al. actualized nipple-sparing mastectomy followed by a direct-to-implant (DTI) reconstruction with prepectoral polyurethane-covered implant positioning with no further coverage. After a mean follow-up of 4 months, no major complications such as BAD or grade III–IV capsular contracture had been observed. Patient satisfaction, assessed using the BREAST-Q, was excellent [11]. In addition, an adequate mastectomy flap thickness and perfusion is the sine qua non condition for successful prepectoral breast reconstruction [39, 40]. Since 2017, Rancati et al. used preoperative digital mammograms to allow an accurate evaluation of the breast coverage, flap quality, and vascularization and reported that only patients with breast subcutaneous tissue coverage above 2 cm would determine an adequate flap to perform a safe immediate DTI reconstruction [26]. Moreover, the thickness of the mastectomy flap has also an impact on the overall satisfaction rate, as seen in the retrospective study of Salgarello et al. [15] where it was reported that the postoperative breast *Q*-scores of satisfaction are encouraging in the use of polyurethane-covered prepectoral implants in immediate breast reconstruction, especially in patients with "thick" mastectomy flaps. Postoperative complications can be reduced by maintaining adequate mastectomy flap thickness and perfusion, as demonstrated by assessment of flap vascularity with ICG and near-infrared imaging. As highlighted by Handel [41], polyurethane foam promotes rapid adherence to surrounding tissues and tissue regeneration by stimulating the formation of a periprosthetic capsule with a three-dimensional architecture, with a random arrangement of collagen fibers. This arrangement of the collagen fibers is not able to produce a linear and uniform vector of contraction, thus reducing the risk of capsular contracture. Furthermore, the extremely adherent texture of this implant reduces the risks of rotation and displacement, and consequently the possible need for revision surgery.

The prepectoral reconstruction with polyurethane-coated implants is spreading more and more with encouraging results, but in the literature there are no studies that have focused on interaction between these implants and PMRT. As is well demonstrated in the literature on implant-based breast reconstruction, adverse effects on breast reconstruction are common when PMRT is required. The main short-term complications are increased risk of seroma, wound dehiscence with prosthetic exposure, and infection, while long-term complications associated with radiotherapy are represented by resistance to expansion, chronic pain, capsular contracture, thinning of the skin that may result in visibility of the prosthetic profile, deformation, and rupture of the implants [42]. Therefore, it is appropriate to discuss, both in a multidisciplinary team and with the patient, the problems and decisions related to the best integration of radiotherapy with the different surgical options. In fact, there are advantages and disadvantages for each type of reconstructive option: with implants (infections, capsular contractures, need for surgical revision and failures), and with flap (fibrosis, volumetric contraction, need for surgical revision) [43–45]. According to the iBAG study, in prepectoral reconstructions with implants and full-coverage porcine ADM (Braxon®), the PMRT appears to be a statistically significant risk factor for the development of capsular contracture [21]. According to another study based of 312 patients, comparing the response to radiotherapy between two patient groups (textured implants vs. polyurethane-covered implants), the relative risk of developing capsular contracture after RT was higher in the textured implant group [14].

In our experience, polyurethane-coated implants may have a protective role against RT damage; the complication rate and the aesthetic outcome obtained from our studies confirm this trend, and in particular, the RT technique delivered seems to play an important role in determining the adverse effects of radiotherapy.

In our study, the group of patients undergoing PMRT with the IMRT technique recorded a higher aesthetic result and less damage from RT (LENT–SOMA score), confirming the advantage of IMRT/VMAT compared to the standard 3D technique [17].

In addition, our results suggest a better cosmetic outcome in patients with thick mastectomy flaps. In fact, we also found a negative linear correlation between the LENT–SOMA score and the thickness of the mastectomy flaps; therefore, we presume that the subcutaneous tissue layer preserved during the mastectomy has a protective role against radiation damage. Furthermore, the study shows that there is a significant correlation between the thickness and the degree of capsular contracture after radiotherapy, confirming that a greater thickness of subcutaneous tissue protects the implant from RT damage. We detected less deformity of the reconstructed breast, presumably due to the absence of the pectoralis major muscle covering the implants, whose radio-induced shortening results in the inframammary fold rising and displacement of the implant [46].

As demonstrated by Rancati, different patients with the same breast volume can present different thicknesses of the subcutaneous tissue covering the mammary gland [26]. Following Rancati's classification, we believe that the best aesthetic results are obtainable in patients with a cutaneous–subcutaneous envelope type 2 (thickness between 1 and 2 cm) or type 3 (thickness greater than 2 cm). The multivariate analysis of our study also shows that an adequate thickness of the mastectomy flaps combined with the IMRT technique allows to obtain less damage from RT (LENT–SOMA score) and therefore a better global aesthetic result of the reconstruction. The main limitations of this study are the retrospective design, the relatively small sample size, and the lack of a control group, which may limit the generalizability of the results. Further prospective studies with larger cohorts are needed to validate these findings.

## Conclusions

Our preliminary data suggest that the RT technique would seem to influence the results after RT and consequently the aesthetic outcome. Also, an adequate thickness of the mastectomy flap allows to obtain more natural shape of the breast with greater patient satisfaction and, in addition, would appear to reduce the negative effects of RT. It is recommended to use polyurethane-coated implant in order to decrease the odds of developing complications. In conclusion, the mechanism that allows this protective action of PU foam is still under study, and further studies with a larger sample are needed to confirm these results.

## Data Availability

Research data are stored in an institutional repository and will be shared upon request to the corresponding author.
